# Exogenous proanthocyanidins improve tolerance of Cu-toxicity by amelioration of oxidative damage and re-programming of gene expression in *Medicago sativa*

**DOI:** 10.1371/journal.pone.0259100

**Published:** 2021-10-26

**Authors:** Siyi Zhao, Yanqiao Zhu, Wenwen Liu, Xiaoshan Wang, Han Wang, Yingping Cao, Fei Chen, Longxing Hu, Lixia Gong, Chunxiang Fu, Zhifei Zhang

**Affiliations:** 1 College of Agronomy, Hunan Agricultural University, Changsha, Hunan, China; 2 Shandong Provincial Key Laboratory of Energy Genetics and CAS Key Laboratory of Biofuels, Qingdao Institute of Bioenergy and Bioprocess Technology, Chinese Academy of Sciences, Qingdao, Shandong, China; 3 Department of Grassland Science, College of Animal Science and Technology, Yangzhou University, Yangzhou City, Jiangsu Province, China; 4 University of Chinese Academy of Sciences, Beijing, China; Nanjing Agricultural University, CHINA

## Abstract

Excess copper (Cu) in soil due to industrial and agricultural practices can result in reduced plant growth. Excess Cu resulted in severely retarded root growth with severe discoloration of Alfalfa (*Medicago sativa*) and *Medicago truncatula*. Growth in the presence of hydrogen peroxide resulted in similar symptoms that could be partially recovered by the addition of the reductant ascorbic acid revealing damage was likely due to oxidative stress. The addition of proanthocyanidins (PAs) in the presence of Cu prevented much of the damage, including plant growth and restoration of lignin synthesis which was inhibited in the presence of excess Cu. Transcriptome analyses of the impact of excess Cu and the amelioration after PAs treatment revealed that changes were enriched in functions associated with the cell wall and extracellular processes, indicating that inhibition of cell wall synthesis was likely the reason for retarded growth. Excess Cu appeared to induce a strong defense response, along with alterations in the expression of a number of genes encoding transcription factors, notably related to ethylene signaling. The addition of PAs greatly reduced this response, and also induced novel genes that likely help ameliorate the effects of excess Cu. These included induction of genes involved in the last step of ascorbic acid biosynthesis and of enzymes involved in cell wall synthesis. Combined, these results show that excess Cu causes severe oxidative stress damage and inhibition of cell wall synthesis, which can be relieved by the addition of PAs.

## Introduction

Copper (Cu) is an essential element that acts as a co-factor for a variety of enzymes involved in various biological processes, including photosynthesis, respiration, reactive oxygen species (ROS) scavenging, cell wall remodeling, and ethylene perception [1,2]. Cu^2+^ ions act as a cofactor in plastocyanin (PC), an essential carrier of electrons in photosynthesis [3], and enzymes such as cytochrome c oxidase, the terminal electron acceptor in respiration [4], laccase, involved in lignin polymerization in plants [5,6], and various other enzymes involved in ROS metabolism such as Cu/Zn superoxide dismutase, ascorbic acid (AsA) oxidase, amine oxidase, and polyphenol oxidase [2].

In the environment, Cu can be found in a wide range of concentrations, from limiting to excess to support optimal plant growth. As a consequence plants have developed various mechanisms to balance cellular Cu levels. Under Cu deficiency, plants modify the acquisition and redistribution of Cu for efficient utilization. In Arabidopsis (*Arabidopsis thaliana*), SPL7 (*SQUAMOSA* promoter binding protein–like7) is a central regulator of Cu homeostasis. SPL7 regulates the expression of genes encoding several Cu transporters including COPT1 and COPT2 (copper transporters), ZIP2 (ZRT/IRT-like protein), FRO3 (ferric reductase oxidase 3), and a Cu chaperone to increase copper uptake under Cu deficiency [7–9]. SPL7 is also required for the expression of several microRNAs, such as miR397, miR398, miR408, and miR857, which are involved in Cu homeostasis [3]. Spatial control of Cu transport is another key aspect of Cu homeostasis. Members of the COPT family such as COPT5 and COPT6 are involved in Cu reallocation when Cu availability decreases. *Copt6* mutants of Arabidopsis exhibit altered Cu distribution by increasing Cu levels in rosette leaves and reducing Cu content in seeds under Cu-deficient conditions. Likewise, *copt5* mutants showed Cu accumulation in the root and decline in siliques and seeds [10,11].

Human activities including mining, intensive use of fertilizers, application of pig and poultry slurries rich in Cu, and the use of Cu-based pesticides have contributed to the increase in Cu content in the soils [2]. A healthy Cu concentration in the soil is ~20–30 mg kg^-1^, but it can reach nearly 100 times higher levels in contaminated soils [12]. Excess Cu is toxic to most plants, causing chlorosis of vegetative tissues, reduced root growth, and defects in floral development and germination [13,14]. At a subcellular level, excess Cu causes a perturbation of photosynthesis and respiration, damage to the plasma membrane, and a wide variety of other metabolic disturbances [14]. Excess Cu also causes a reduction in the absorption of macro and micronutrients, including nitrogen, phosphorus, calcium, magnesium, potassium, zinc, and manganese [14,15]. Cu toxicity is accompanied by the formation of ROS, such as hydrogen peroxide (H_2_O_2_), superoxide anion radical (O_2_^•−^), and hydroxyl radical (HO•). Over accumulation of ROS can cause oxidation of proteins, membrane fatty acids, and DNA, resulting in cellular damage and death [16]. To combat the detrimental effects of exposure to excess Cu, plants have evolved detoxification mechanisms that are mainly based on chelation, subcellular compartmentalization, and antioxidant systems. Chelation of Cu is a ubiquitous detoxification strategy in a wide variety of plants. Phytochelatins and phenolics have a high binding affinity to Cu. Phytochelatins form complexes with heavy metal ions and transport them from the cytosol into the vacuole [17]. In maize, induction of phytochelatins starts very soon after exposure to Cu and cadmium (Cd) [18]. Antioxidant systems, including superoxide dismutase (SOD), peroxidase (POD), ascorbate peroxidase (APX), catalase (CAT), and AsA-glutathione cycle, play an important role in protecting plants via ROS scavenging. In this system, dismutation of O_2_^•−^ to H_2_O_2_ by SOD is the primary line of defense. Degradation of H_2_O_2_ to H_2_O is catalyzed by CAT, POD, and APX [19,20]. Activities of antioxidant enzymes in different plant species increase under Cu treatment [13,21–23].

Proanthocyanidins (PAs) are the oligomers or polymers of flavan-3-ol units, and the second most abundant polyphenolic compounds in plants after lignin [24]. PAs are synthesized via the flavonoid and the phenylpropanoid pathways and present multiple biological functions, such as protecting plants against pathogen attack and predation, and inhibiting the growth of neighboring plants [25,26]. As metal ion chelators, PAs form stable complexes with metal ions, which influence their bioavailability [27]. PAs are natural polyphenolic antioxidants that are widely available in plants, especially in fruits and seeds [28,29], and show excellent protection against free radicals with greater reductant capacity than AsA, tocopherol E, and β-carotene [30]. In Arabidopsis, the germination of PA-deficient mutant seeds was inhibited under oxidative stress conditions, suggesting a loss of PA-related anti-oxidative ability [31]. PA-deficient mutants also exhibit reduced dormancy and faster deterioration during natural aging at room temperature [32], and ROS damage is considered as the key factor inducing seed deterioration. In forage crops, the presence of PAs is an important agronomic trait. PAs bind to proteins in the rumen and reduce the rate of fermentation and methane production, through which more intact proteins to exit the rumen, thus improving nitrogen nutrition [33]. In addition, cucumber seedlings pretreated with exogenous PAs suffered less damage by the abiotic stress, such as high radiation, polyethylene glycol, and cold stress maintained higher content of chlorophyll, abscisic acid, and alternative oxidase protein [34].

Alfalfa (*Medicago sativa*) is an important perennial leguminous forage species of high nutritional value to livestock. Alfalfa has a good ability to withstand heavy metal toxicity, including that caused by Cd, Ni, Cu, and Hg; most of the information on this response of alfalfa has been confined to studies on antioxidant defense systems [35–38]. This work was carried out to investigate the changes in seedling growth, physiology and oxidative stress in alfalfa plants in response to Cu toxicity and the associated potentially protective mechanisms of PAs. We also carried out transcriptome analysis in *Medicago truncatula* to examine the changes that occurred due to Cu toxicity and its amelioration by PAs.

## Material and methods

### Seed germination test

Seeds of two alfalfa cultivars, WL656 and Southern Hemisphere (SH), were obtained from Hunan Agricultural University, Changsha, China. Seeds were sterilized in 5% (v/v) sodium hypochlorite for 10 min and rinsed 5–6 times with distilled water. Seeds were germinated on sterile filter paper under 16 h light/8 h dark at 25 ± 2 °C, and 60% relative humidity. For pretreatments with PAs and AsA, 5 ml of different concentrations PAs (0, 50, 100, 150, 200, 250 mg L^-1^) and different concentrations of AsA (0, 50, 100, and 150 mg L^-1^) were used, respectively, as germination medium for the first 2 d. For Cu and H_2_O_2_ treatments, 5 ml of different concentrations of CuSO_4_•5H_2_O (0, 25, 50, 100, 200 and 400 mg L^-1^ CuSO_4_•5H_2_O) or different concentrations of H_2_O_2_ (0, 90,180, and 450 mM) were added after 2 d of germination of seeds that were pretreated with PA, AsA, or H_2_O. Distilled water was used as the control treatment. Seed germination rate, root length, and root dry weight were measured after 10 d of germination. Each treatment was carried out in triplicates.

The seeds of *Medicago truncatula* (R108) were polished with sandpaper to promote germination. These seeds were pretreated with 100 mg L^-1^ PAs or H_2_O for 2 d and then changed to 100 mg L^-1^ Cu. Seed germination rate and root length were measured after 10 d of germination. Each treatment was carried out in triplicates.

### Detection of H_2_O_2_ by 3,3-diaminobenzidine (DAB) staining

Detection of H_2_O_2_ by DAB staining was carried out as previously described [39]. Ten-day-old WL656 and SH seedlings were immersed in DAB staining solution (1 mg mL^-1^, pH = 5.8), incubated in vacuum for 10 min, and then kept on a shaker for 5 h at 80 rpm in the dark. Samples were then decolorized in 80% ethanol in a boiling water bath. After decoloration, samples were treated with 100% ethanol and then kept at 4 °C for 4 d.

### RNA isolation and qRT-PCR

Total RNA was extracted from the plant tissues using a TRIzol kit (TransGen Biotech, Beijing, China) and was reverse transcribed into cDNA using a PrimeScript^™^ RT Reagent Kit with gDNA Eraser (Takara, Dalian, China) according to the manufacturers’ instructions. The qRT-PCR analysis was performed in a 20 μL reaction volume containing 10 μL of SYBR Premix ExTaq^™^ (Takara, Dalian, China), 2 μL of cDNA, and 0.5 μM of each primer. The primer pairs used for qRT-PCR are listed in S1 Table. *ACTIN* was used as a reference for normalization. The cycle thresholds were determined using a Roche Light Cycler 480 II sequence detection system (Roche, Shanghai, China).

### RNA seq analysis

RNA-seq libraries were performed using the TruSeq Stranded mRNA Library Prep Kit according to the manufacturer’s instructions (Illumina) with total RNA isolated as described above, and sequenced on a NextSeq500 system (Illumina) with an average quality score (Q30) of above 95%. Quality control was carried by using FastQC software (https://www.bioinformatics.babraham.ac.uk/projects/fastqc/). A paired two-tailed *t*-test on the FPKM (per kilobase per million mapped reads) [40] values for each comparison, that is, Cu/control and PAs/control, was conducted. Genes with *p*-value < 0.05 and |Log_2_FC|≥1 were considered significant in each comparison. Fold change was calculated by comparing Cu/control and PA/control. Log_2_FC was calculated by taking log_2_ of the calculated fold change. Genes with FPKM values equal to zero for both replicates in a treatment were not considered in the analysis. Genes that passed the *p*-value cut off and FPKM threshold in each comparison were used for Venn diagram construction. GO (Gene Ontology) term enrichment analysis was performed on selected overlap groups using the ClueGO plugin for Cytoscape [41]. RNA-seq raw data were deposited at the National Center for Biotechnology Information Sequence Read Archive database under project identifier PRJNA734715.

### Histochemical staining of lignin

Histochemical staining of lignin was carried out according to the method described previously [42]. The stem segment of *Medicago truncatula* samples was sectioned manually using a razor blade. Isolated sections were stained in 5% phloroglucinol staining solution at room temperature for 3 min. Images were acquired using a fluorescence microscope (Olympus BX51, Japan).

### Statistical analysis

The data were statistically analyzed using SPSS software (Chicago, USA), and the significance was calculated based on the one-way ANOVA test of three biological replicates. The difference was a significant difference when *p* ≤ 0.

## Results

### PAs improved the tolerance of alfalfa seedling exposed to excess Cu

**Fig 1:**
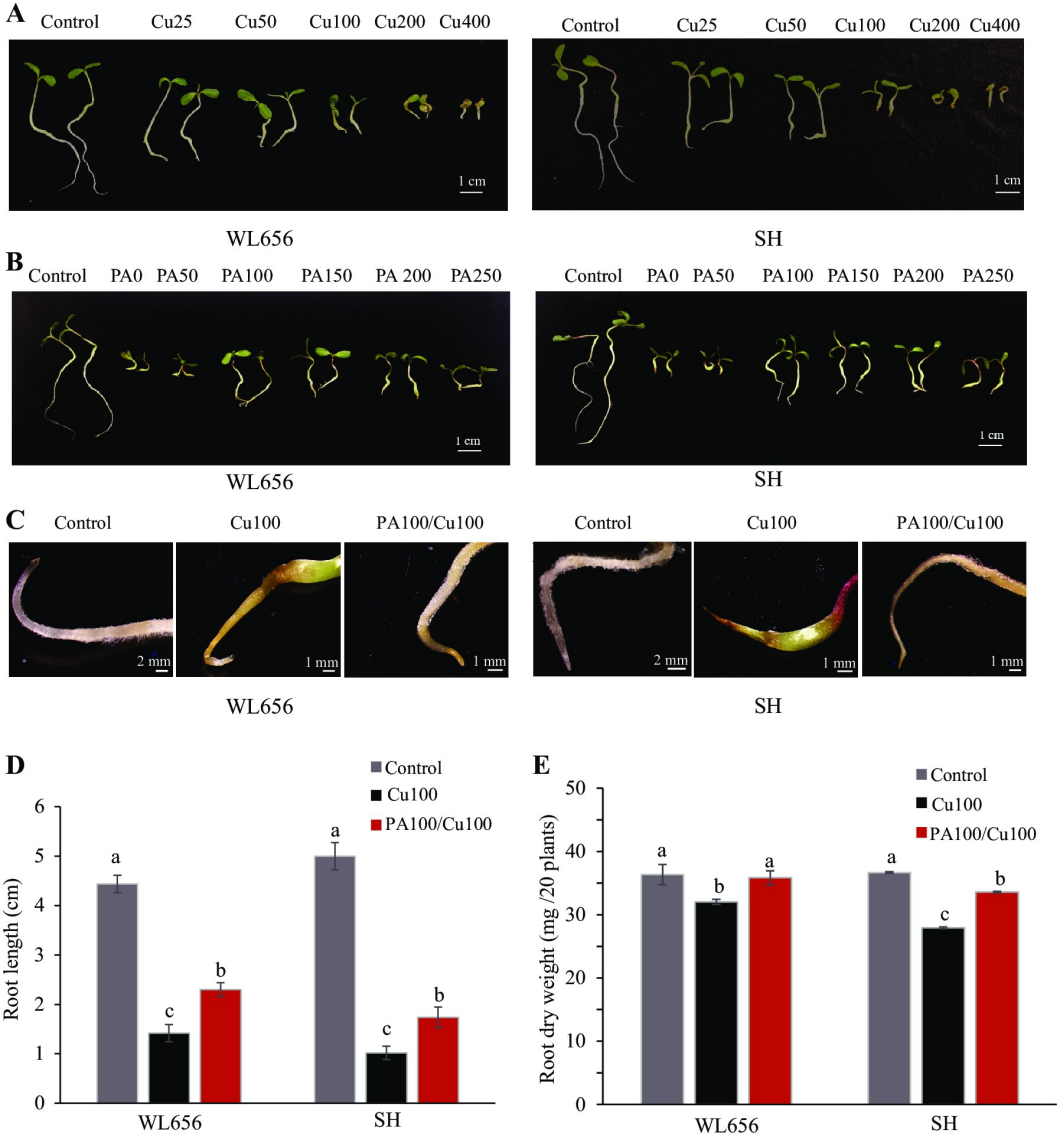
Proanthocyanidins (PAs) improved alfalfa seedling growth under excess Cu conditions. (A) The effect of different concentrations of Cu on seedling growth. (B) The effect of different concentrations of PA pretreatment on seedling growth with excess Cu treatment. PAs alleviated Cu toxicity on root status (C), increased root length (D) and root dry weight (E) under Cu stress. Cu 25, 50, 100, 200, 400 represents 25, 50, 100, 200, 400 mg L^-1^ CuSO_4_•5H_2_O. PA0, 50, 100, 150, 200, 250 represents 0, 50, 100, 150, 200, 250 mg L^-1^ PAs, respectively. Means ± SE of three biological replicates. Significant differences (*p* ≤ 0.05) were denoted by different lowercase letters.

The effects of PA pretreatment on high Cu toxicity on alfalfa were assessed by using WL656 and SH cultivars. Two days old seedlings were treated with medium containing 25 to 400 mg L^-1^ CuSO_4_•5H_2_O (defined as Cu25 to Cu400). Cu toxicity symptoms appeared on roots grown with Cu25 to Cu100 medium (Fig 1A). Root Growth was severely inhibited in Cu200 and Cu400. Leaves underwent browning or senescence when Cu concentration was over 100 mg L^-1^. Therefore, 100Cu was chosen for Cu treatment but did not cause lethality (Fig 1A and S1 Fig). Cu toxicity was alleviated when seeds were pretreated with different concentration of PAs (from 50 to 250 mg L^-1^, defined as PA50 to PA250) for both cultivars WL656 and SH (Fig 1B), and PA100 showed optimum effects against Cu toxicity. Root growth was inhibited when PA concentration was greater than 200 mg L^-1^ (Fig 1B). The combination of Cu100 and PA100 were chosen for further experiments. WL656 and SH had pale roots with abundant root hair (Fig 1C). After exposure to excess Cu, the phenotype of root exhibited growth retardation, the roots changed to brown or green and were shorter without any visible root hair. PAs pretreatment partially mitigated Cu toxicity in the roots, although the root status did not recover to that of the control level. Roots remained whitish or light brown with observable root hair formation (Fig 1C). While Cu treatment significantly (P < 0.05) decreased root length and root dry weight by ~60% and ~80%, respectively, PA treatment significantly (P < 0.05) improved root length and root dry weight (Fig 1D and 1E).

### PAs improved the performance of alfalfa seedling under oxidative stress

**Fig 2:**
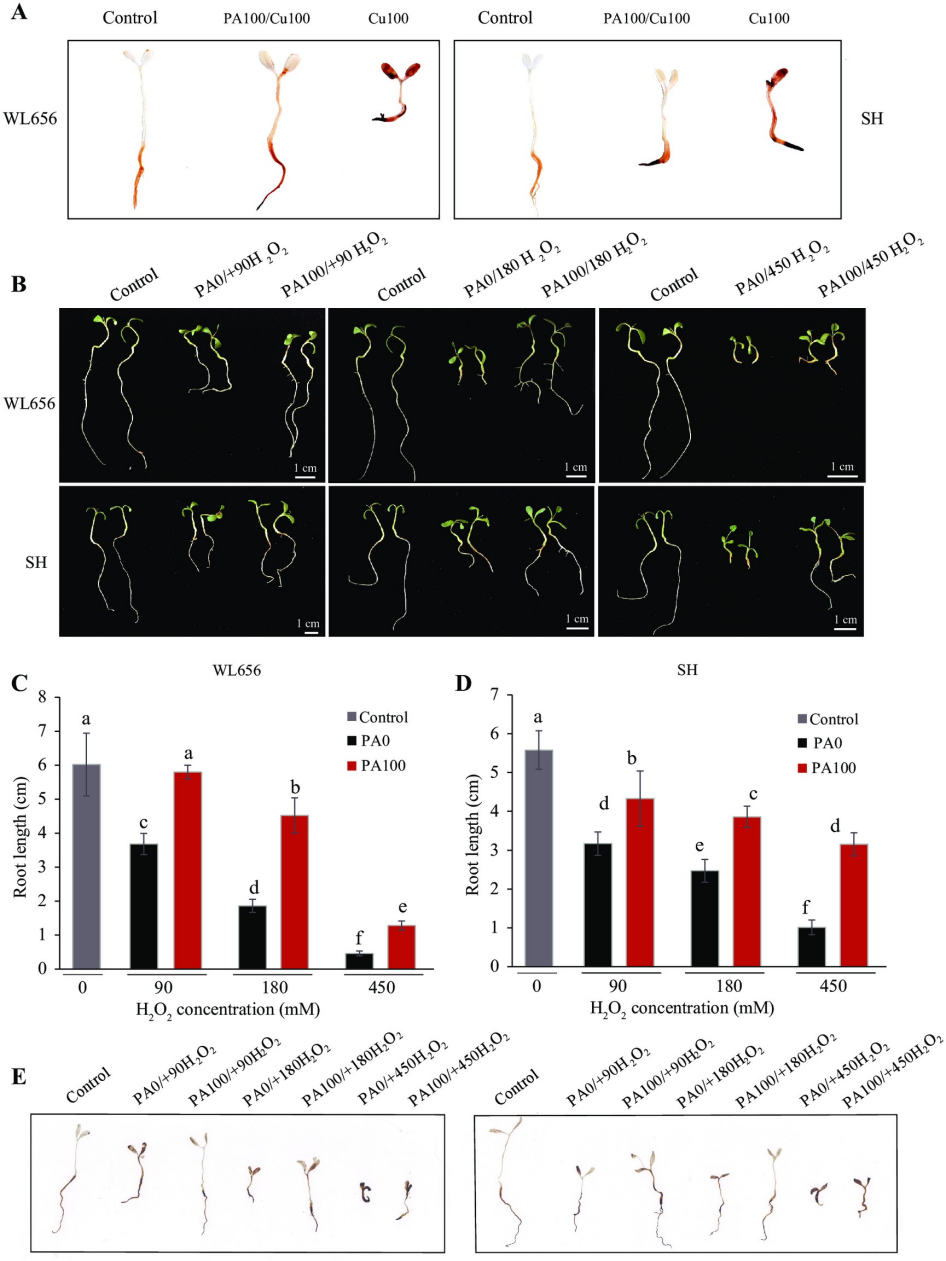
Proanthocyanidins (PAs) improved alfalfa seedling growth under oxidative stress. (A) Histochemical staining with DAB under Cu stress pretreated with PAs. (B) The phenotype of seedlings after 8 d of excess H_2_O_2_ treatment or pretreated with PAs. PAs increased the root length of WL656 (C) and SH (D) under excess H_2_O_2_ treatment. (E) Histochemical staining with DAB under different concentration of H_2_O_2_ or pretreated with PAs. PA0: 0 mg L^-1^ PAs pretreatment, PA100: 100 mg L^-1^ PAs pretreatment, Cu100: 100 mg L^-1^ CuSO_4_•5H_2_O, 90 H_2_O_2_: 90 mM H_2_O_2_, 180 H_2_O_2_: 180 mM H_2_O_2_, 450 H_2_O_2_: 450 mM H_2_O_2_. Means ± SE of three biological replicates. Significant differences (*p*≤ 0.05) were denoted by different lowercase letters.

The effect of excess Cu stress and PAs treatments on ROS generation in alfalfa plants was assessed by *in vivo* histochemical staining with DAB. There was an increase in the formation of dark brown lesions on leaves and roots, especially the root tips, after exposure to Cu stress (Fig 2A and S2 Fig). When plants were pretreated with PAs, the dark brown lesions reduced in leaves and roots, and the color of leaves almost recovered to that observed in the control (Fig 2A and S2 Fig).

Seedling development was inhibited after exposure to an increasing concentration of H_2_O_2_ from 90 mM to 450 mM. The phenotype of plants treated with 450 mM H_2_O_2_ was very similar to those treated with Cu100, with severely inhibited root development (Fig 2B). Root length decreased progressively with increasing H_2_O_2_ stress. Under minor H_2_O_2_ stress (90 mM H_2_O_2_), the root length of plants recovered to that of the control after PAs pretreatment. However, the root length of plants under 450 mM H_2_O_2_ treatment recovered less than 20% compared to that of the control. PAs treatment significantly (P < 0.05) improved root length for both WL656 and SH treated with different concentrations of H_2_O_2_ (Fig 2C and 2D). Analysis of DAB staining revealed that dark brown lesions increased with increasing H_2_O_2_ concentrations. PAs reduced the formation of dark brown lesions, especially under high H_2_O_2_ stress (Fig 2E). Thus, PAs may protect alfalfa seedlings from Cu toxicity by eliminating ROS such as H_2_O_2_ produced due to excess Cu.

### PAs alleviated oxidative stress induced by excess Cu by its antioxidant capacity

**Fig 3:**
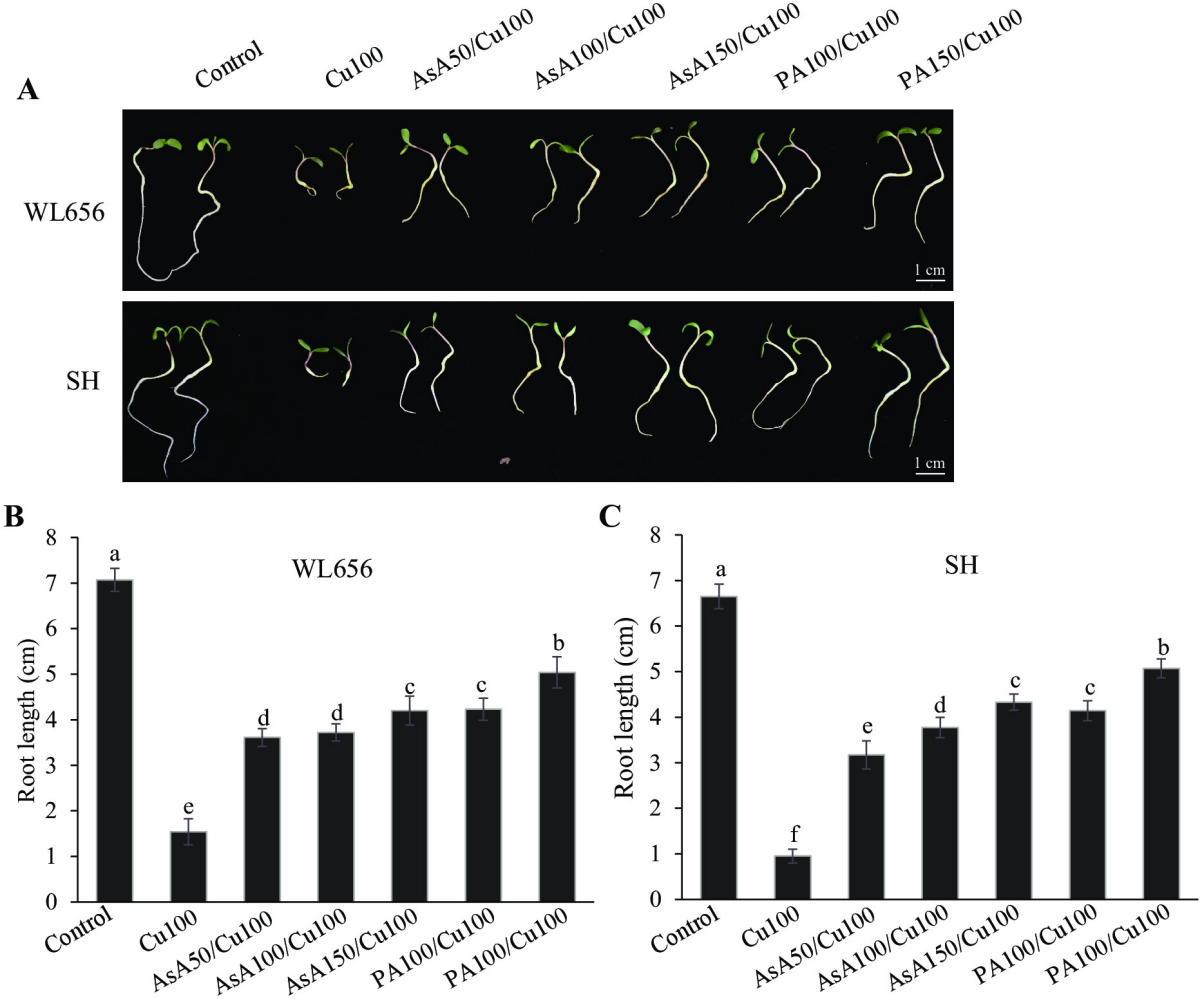
Ascorbic acid (AsA) improved alfalfa seedling growth under excess Cu conditions. (A) The phenotype of seedlings after 8 d of excess Cu treatment or pretreated with different concentrations of AsA. AsA increased the root length of WL656 (B) and SH (C) under Cu stress. Cu100: 100 mg L^-1^ CuSO_4_•5H_2_O, AsA50: 50 mg L^-1^ AsA pretreatment, AsA100: 100 mg L^-1^ AsA pretreatment, AsA150: 150 mg L^-1^ AsA pretreatment, PA100: 100 mg L^-1^ PAs pretreatment, PA150: 150 mg L^-1^ PAs pretreatment. Means ± SE of three biological replicates. Significant differences (*p*≤ 0.05) are denoted by different lowercase letters.

AsA is an excellent antioxidant that protects plants from oxidative damages via its ROS scavenging capability [37]. Different concentrations of AsA from 50 to 150 mg L^-1^ were used to treat both WL656 and SH before treating with excess Cu. AsA improved the performance of alfalfa seedling at each concentration with observable white and elongated roots and reduced DAB staining (Fig 3A and S2 Fig). Likewise, pretreatment with both 100 and 150 mg L^-1^ PAs showed a similar protective effect as did AsA against Cu stress. AsA treatment significantly (P < 0.05) increased root length under Cu stress, and the effect was more significant (P < 0.05) when treated with 150 mg L^-1^ AsA than with 50 or 100 mg L^-1^ AsA. PAs treatment at the same concentration showed significantly increased root length than that after AsA treatment (Fig 3B and 3C). Therefore, PAs may act as a strong reductant to improve the tolerance of alfalfa seedlings to high Cu stress.

### PAs improved the growth rate of *Medicago truncatula* (R108) seedling under Cu stress

**Fig 4:**
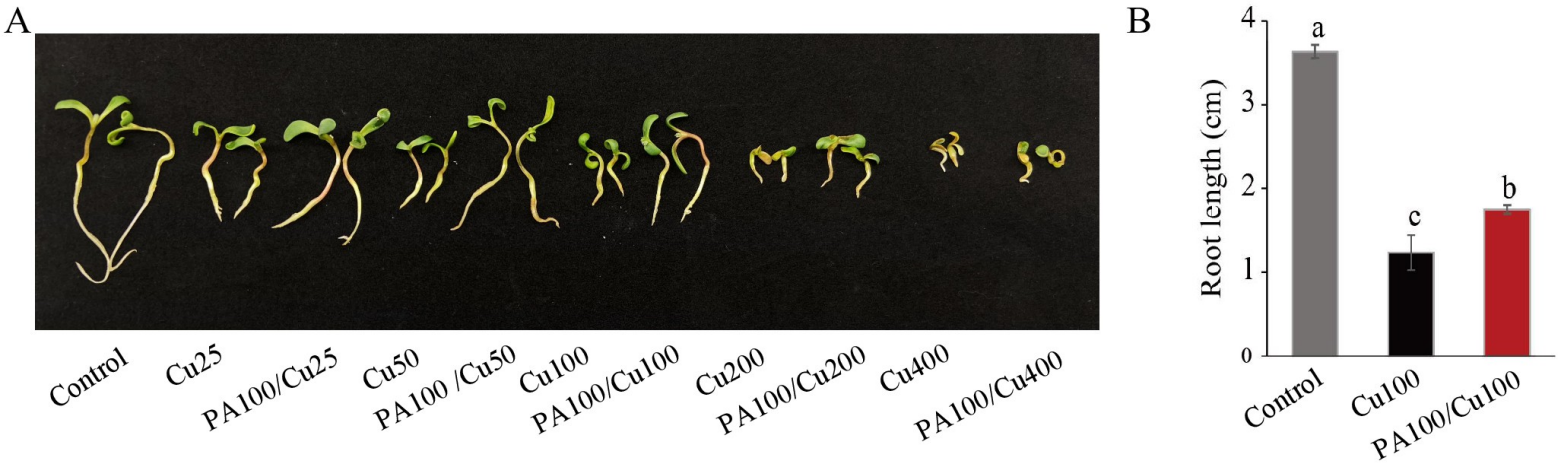
Proanthocyanidins (PAs) improved *Medicago truncatula* (R108) seedling growth under excess Cu conditions. (A) The phenotype of seedlings after 8 d of different concentrations of Cu treatment or pretreated with PAs. (B) PAs increased root length under Cu stress (n = 20). Cu 25, 50, 100, 200, 400 represents 25, 50, 100, 200, 400 mg L^-1^ CuSO_4_•5H_2_O, respectively. PA100: 100 mg L^-1^ PAs pretreatment. Means ± SE of three biological replicates. Significant differences (*p*≤ 0.05) are denoted by different lowercase letters.

Alfalfa is autotetraploid and the genomes of different cultivars vary. Although the alfalfa genome has been sequenced, the genomic information is still emerging [43,44]. To explore the underlying mechanism of PAs-mediated protection in alfalfa, we choose *Medicago truncatula* R108 for further studies. PAs showed similar protection in R108 seedling development compared to two alfalfa cultivars, WH656 and SH. Seedling growth and root length in these cultivars were severely inhibited after exposure to different concentrations of Cu compared to those in the control (Fig 4A). Seedlings pretreated with 100 mg L^-1^ PAs could alleviate Cu toxicity with elongated root length (Fig 4B).

### Differentially expressed genes in *Medicago truncatula* R108 under high Cu stress

**Fig 5:**
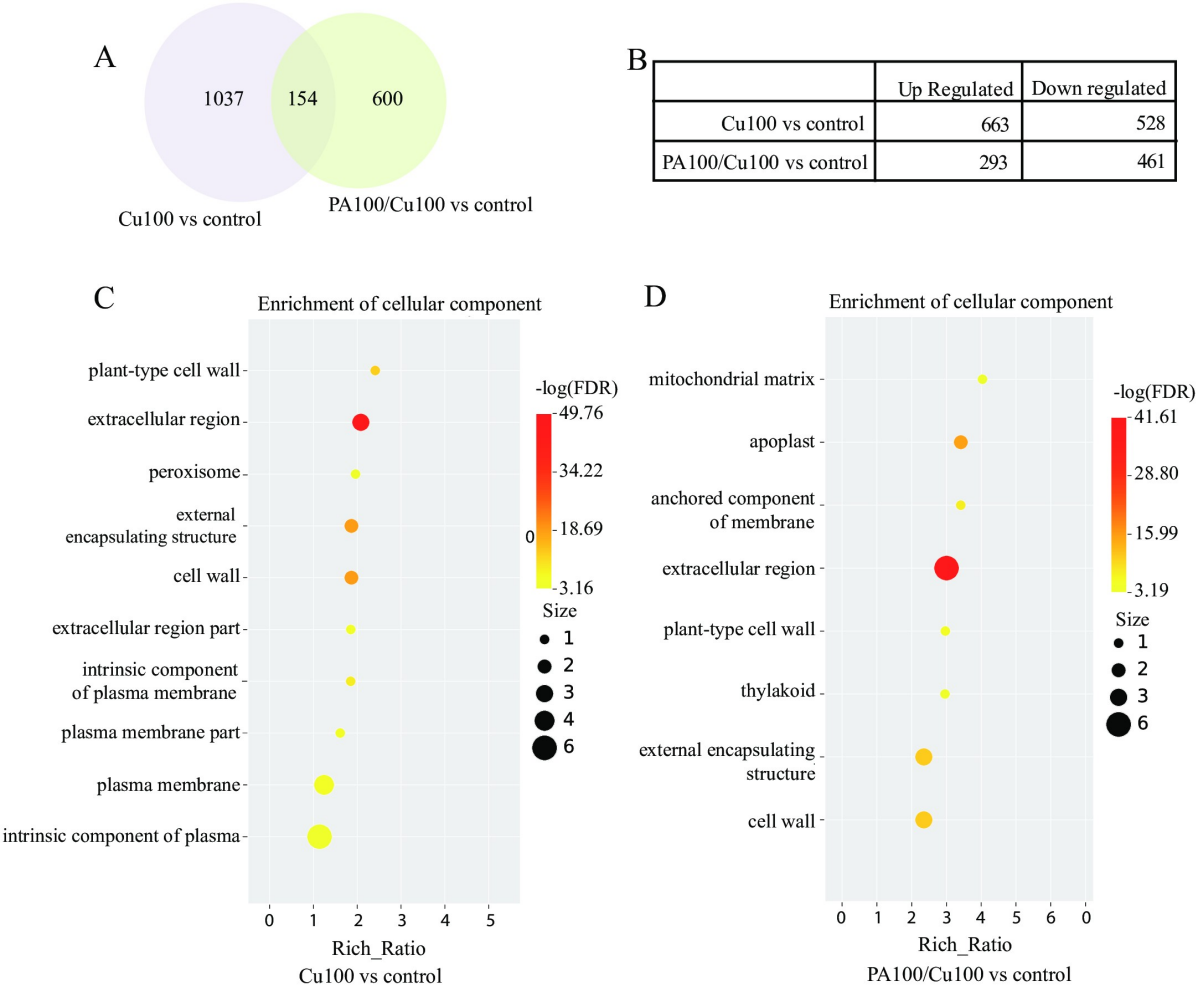
Analysis of differentially expressed genes (DEGs) under Cu stress. (A) Venn diagram overlap of DEGs between different comparisons. (B) Number of up-regulated and down-regulated genes in different comparisons. (C) Cellular component enrichment of DEGs under Cu stress. (D) Cellular component enrichment of DEGs under Cu stress pretreated with PAs. The bubble diagram in (C) and (D) shows the top 10 Gene Ontology items with the highest enrichment significance. The bigger bubble, higher rich-ratio, and red color indicate a higher enrichment degree. If there are fewer than 10 items, all items will be displayed. PA100: 100 mg L^-1^ PAs pretreatment, Cu100: 100 mg L^-1^ CuSO_4_•5H_2_O.

RNAseq analysis was carried out to compare the effect of Cu treatment with or without PAs pretreatment with that in the control. A paired two-tailed *t*-test was conducted on the FPKM values for different comparisons. Gene expressions changed two-fold and *p*-value < 0.05 was considered significant in each comparison. A total of 1,791 differentially expressed genes (DEGs) were detected, among which 1,037 DEGs were observed only in plants under excess Cu treatment, 600 DEGs were detected only in plants pretreated with PAs, and 154 DEGs changed under both treatments (Fig 5A, S2 Table). Treatment with excess Cu induced more DEGs compared to PAs pretreatment, with 663 up-regulated, and 528 down-regulated DEGs. Plants pretreated with PAs (+ PAs + Cu) under Cu stress showed 293 up-regulated and 461 down-regulated DEGs (Fig 5B). Excess Cu induced a greater than a two-fold increase in the expression of over 398 genes, and in the majority of the cases this was reduced after PAs pretreatment, indicating the ability of PAs to prevent the changes in gene expression. Many of the induced genes were related to pathogen defense so that excess Cu could trigger a defense response, in agreement with previous reports in Arabidopsis of activation of ethylene and salicylate defense pathways due to excess Cu [45].

Enrichment analysis of DEGs for cellular components showed that genes related to the extracellular region, an intrinsic component of plasma, plasma membrane, and cell wall were more enriched in response to excess Cu treatment. However, more enriched DEGs were related to the extracellular region, external encapsulating structure, and cell wall when plants were pretreated with PAs (Fig 5C and 5D). Molecular function enrichment analysis showed that genes related to antioxidant activity were enriched in response to Cu treatment (S3 Fig). The transcript expression levels of genes involved in antioxidant activity were tested in both alfalfa cultivars WL656 and SH (S4 Fig). The relative transcript levels of manganese/iron superoxide dismutase (*MsG0380016734*), copper/zinc superoxide dismutase (*MsG0680031329*), Catalase (*MsG0380017935*) and Glutathione reductase (*MsG0680031828*) were all up-regulated under Cu treatment, this was reduced after PAs treatment, indicating that PAs likely decreased the oxidative stress, consistent with the above-mentioned physiology.

**Fig 6:**
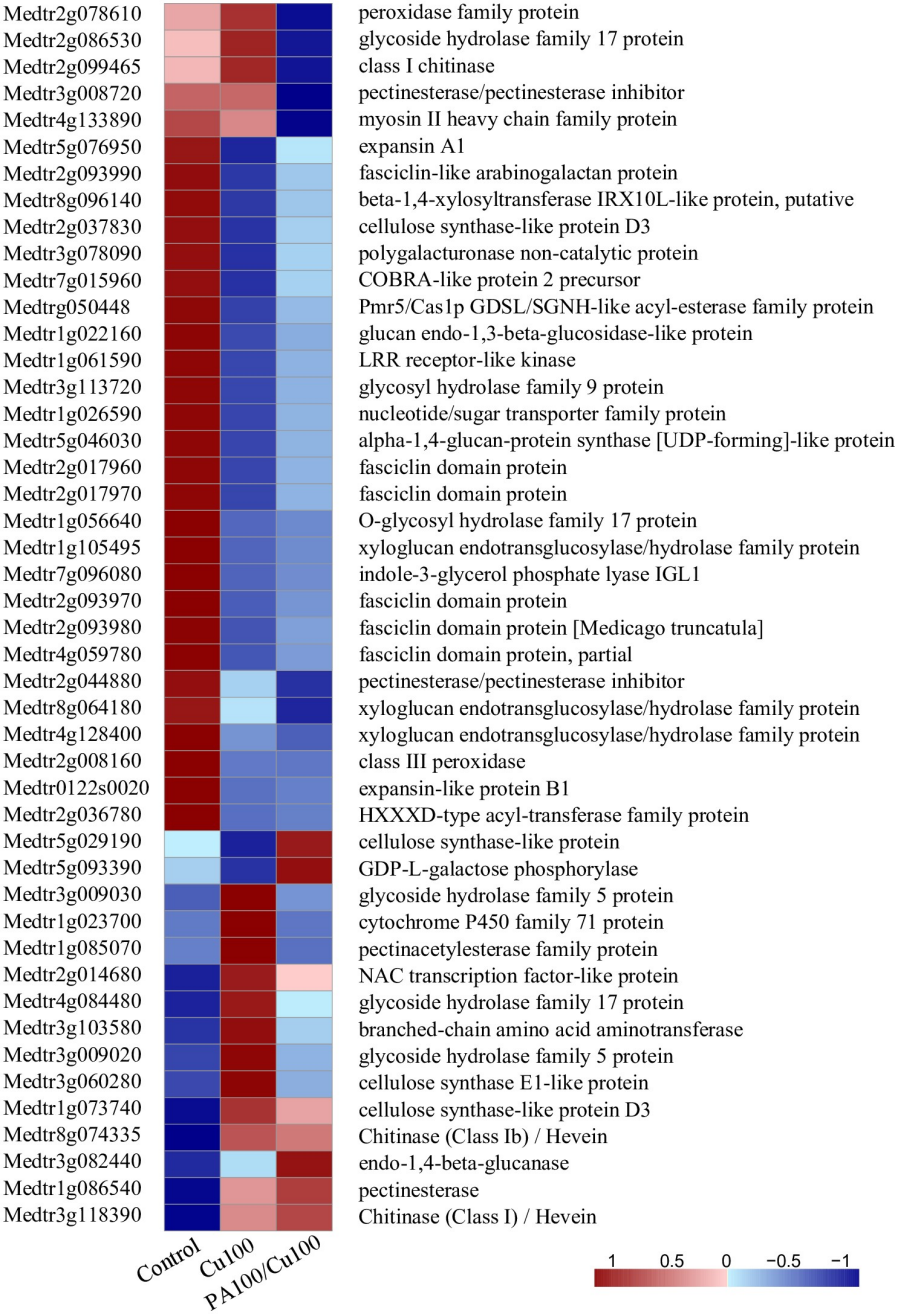
Heatmap shows the expression profile of differentially expressed genes in the cell wall and represents Z-scores of transcripts per million (TPM) for each gene. PA100: 100 mg L^-1^ PAs pretreatment, Cu100: 100 mg L^-1^ CuSO_4_•5H_2_O.

The growth stage and ROS level indicated that PAs pre-treatment could ameliorate Cu toxicity. A high accumulation of ROS was reported to signal initiation of cell wall formation [46]. Therefore, we investigated whether this had any effect on genes associated with the cell wall. RNA-seq data revealed changes in the expression of a variety of genes and most of them were associated with cell wall or extracellular processes (Fig 5C). It was apparent that although the addition of PAs could rescue growth in the presence of Cu at a toxic level, it did not restore the status of seedlings to the normal growth path (Fig 6, S3 Table). Examination of the genes encoding proteins associated with cell wall synthesis revealed that the pattern of PAs pretreatment was not similar to that in wild type or the control. Thus, 26 genes that control cell wall components were down-regulated after Cu treatment, still remained low after PAs pretreatment (Fig 6); in fact, five more genes, including peroxidase family protein, glycoside hydrolase family protein 17, class I chitinase, pectinesterase inhibitor, and myosin II heavy chain family protein were down-regulated with PAs and Cu treatment compared to Cu alone (Fig 6). The expression of two genes associated with cell wall increased after PAs and Cu treatment—cellulose synthase-like protein and GDP-L-galactose phosphorylase, the latter is a key enzyme that catalyzes the last step of ascorbic synthesis in plants [47,48]. Thus, PAs treatment appeared to up-regulate the synthesis of a crucial anti-oxidant, AsA, which, as shown above, can ameliorate the toxic effects of Cu toxicity (Fig 3). Also, endo-1,4-beta-glucanase (MTR_3g0822440), which plays a key role in cell wall synthesis and growth, exhibited notable up-regulation. The increase here after PAs and Cu treatment, compared to both untreated and only Cu-treated samples suggests that an additional enzyme is activated to drive growth [49]. Furthermore, the induction of glycoside hydrolase family 17 protein and chitinase with Cu-treatment reduced significantly in the presence of PAs, and the breakdown of the cell wall was prevented [50]. Finally, the large increase in the expression of a gene encoding myosin after PA and Cu treatment plays a role in the exocytosis of cellulose synthase complexes [51]. Relative transcript expression levels of eight orthologous genes encoding proteins associated with cell wall synthesis were tested in alfalfa cultivars WL656 and SH, including cellulose synthase-like protein D3, GDP-L-galactose phosphorylase, Chitinase (Class Ib) / Hevein, Chitinase (Class I) / Hevein, cellulose synthase E1-like protein, glycoside hydrolase family 17 protein, cytochrome P450 family 71 protein, endo-1,4-beta-glucanase. Transcripts in alfalfa showed similar changes as transcripts in *Medicago truncatula*, confirming that cell wall synthesis was disturbed after Cu treatment, and PA alleviated Cu toxicity in cell wall synthesis (S5 Fig).

In terms of transcription factors, several ethylene AP2 type transcription factors changed in abundance with excess Cu, including Medtr1g098460 and Medtr7g055857 by greater than five-fold that was ameliorated by PAs (S4 Table). There are conflicting reports about ethylene induction in Cu toxicity [52,53], but it appears that at least some factors related to ethylene signalling are altered, but this may be due to alterations of either ABA or auxin, that have also been reported to be involved in Cu toxicity. A TCP (Teosinte branched1/Cincinnata/proliferating)-like transcription factor was also induced by more than five-fold and was reduced after PAs treatment. TCP transcription factors can reduce meristem activity and resulting growth [54]. Almost three times the amount of transcription factors were altered after PAs and Cu treatment compared to that after Cu treatment alone. A variety of other transcription factors were also induced by Cu, including a negative regulator of transcription (Medtr4g070340) and a TAZ domain transcription factor (Medtr5g015680), that interacts with a variety of other transcription factors.

### PAs improved root lignin accumulation under Cu stress

**Fig 7:**
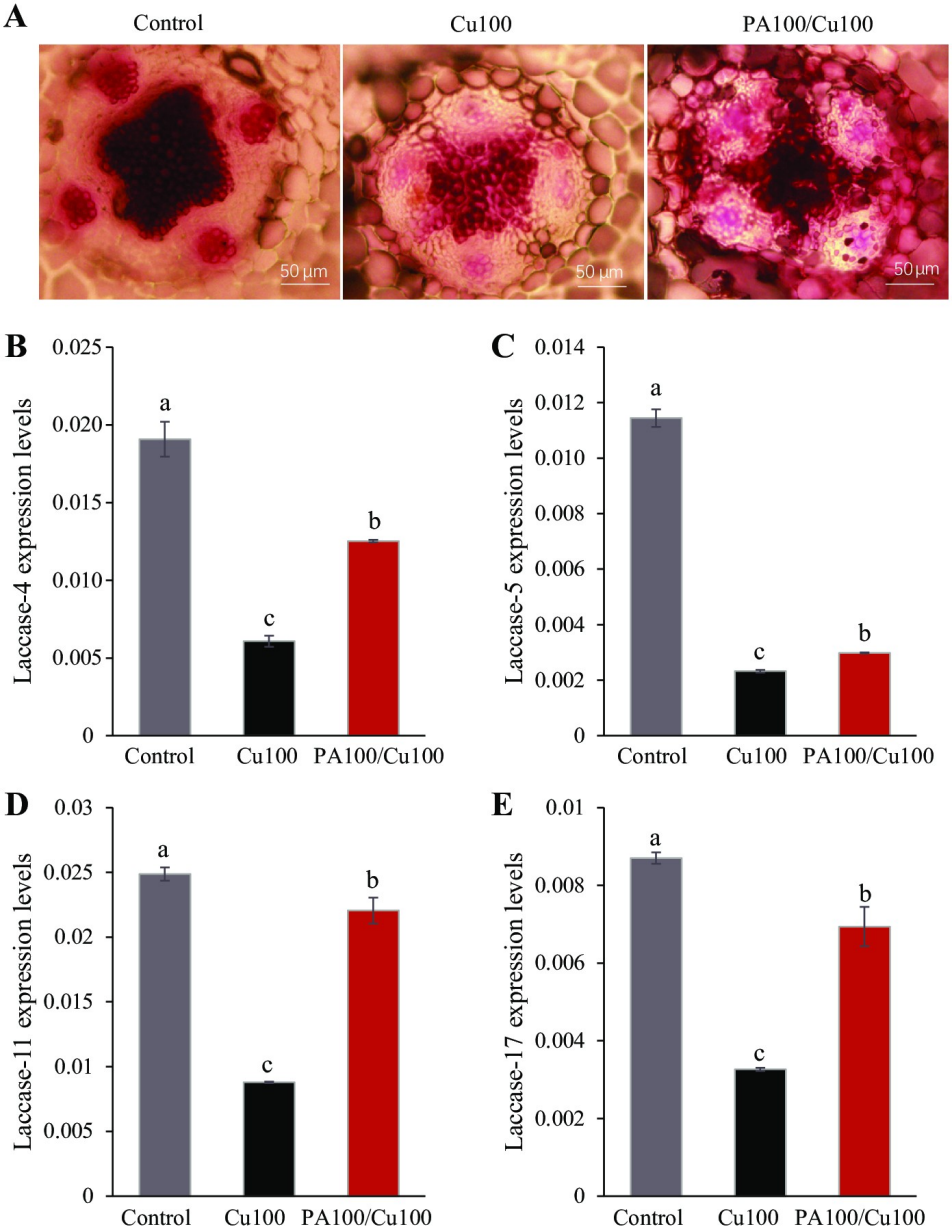
Proanthocyanidins (PAs) improved root lignin accumulation in R108 under Cu stress. (A) Phloroglucinol staining results of roots under Cu stress and PAs pretreatment. Transcriptional expression level of (B) MtrLAC-4, (C) MtrLAC-5, (D) MtrLAC-11, and (E) MtrLAC-17. PA100: 100 mg L^-1^ PAs pretreatment, Cu100: 100 mg L^-1^ CuSO_4_•5H_2_O. Means ± SE of three biological replicates. Significant differences (P ≤ 0.05) are denoted by different lowercase letters.

Phloroglucinol staining of R108 roots showed a decrease in lignin accumulation under Cu stress. Pretreatment of PAs alleviated this effect of excess Cu toxicity on lignin accumulation. Lignin is a polyphenolic polymer that is generated by the oxidation of ROS-dependent peroxidases and laccases [55]. Simultaneous disruption of AtLAC11 (laccase 11), AtLAC4, and AtLAC17 causes severely abolished lignin deposition in Arabidopsis roots [56]. In this experiment, we detected the transcriptional expression level of four isoforms of laccases MtrLAC-4 (Medtr3g462760), MtrLAC-5 (Medtr5g083360), MtrLAC-11 (Medtr5g020600), and MtrLAC-17 (Medtr7g458880). MtrLAC-4, MtrLAC-5, MtrLAC-11, and MtrLAC-17 are homologous to AtLAC-4 (AT2G38080), AtLAC-5 (AT2G40370), AtLAC-11 (AT5G03260), and AtLAC-17 (AT5G60020), respectively, in Arabidopsis (S6 Fig). The transcriptional expression levels of all four isoforms of laccases in R108 reduced significantly (P < 0.05) under Cu stress and the levels increased significantly (P < 0.05) when seedlings were pretreated with PAs. Transcript abundance of MtrLAC-4, MtrLAC-5, MtrLAC-11, and MtrLAC-17 increased more than two-fold with PA pretreatment under Cu stress compared to that in excess Cu treatment alone (Fig 7).

## Discussion

Cu toxicity in the soil can result in a reduction of plant germination and growth, and also pose a threat to human health as excess Cu levels may accumulate in food. This study reveals that excess Cu resulted in severe oxidative stress leading to retarded growth and development, similar to that was observed after H_2_O_2_ was added, and these symptoms could be partially relieved upon the addition of AsA and PAs. Oxidative stress occurred in seedlings exposed to excess Cu and it was expressed as considerably greater staining of H_2_O_2_ than in control plants. H_2_O_2_ is an important redox molecule as it is remarkably stable (half-life > 1 ms) in plant cells [57]. H_2_O_2_ can be transported by cell membrane aquaporins and react with DNA and proteins by oxidation of cysteine and methionine residues, causing long-distance oxidative damage [57,58]. Enhanced levels of H_2_O_2_ were also observed in wheat (*Triticum aestivum* L.), rice (*Oryza sativa* L.), and *Arabidopsis thaliana* under Cu stress [21,59,60]. In this study, alfalfa seedlings treated with a high concentration of H_2_O_2_ showed a similar phenotype as that after excess Cu treatment, indicating that Cu toxicity in plants was induced partially by H_2_O_2_ oxidative damage. PAs pretreatment increased alfalfa seed germination, enhanced healthy root development, and root hair formation, demonstrating the ameliorative role of PAs in reducing Cu toxicity. On the other hand, PAs pretreatments showed similar protective effects on alfalfa plants as those by AsA under Cu stress. AsA, the most abundant non-enzymatic antioxidant in plants, is oxidized to monodehydroascorbate by the reaction catalyzed by ascorbate peroxidase, and thus detoxifies oxidative damage caused by H_2_O_2_ [61,62]. Increasing concentration of PAs and AsA alleviated Cu toxicity on alfalfa plants by increasing root length, and PAs showed better performance than AsA at the same concentrations, indicating that PAs is a powerful antioxidant. DAB staining also confirmed that PAs reduced the accumulation of H_2_O_2_ in the root and leaf. The results indicate that Cu stress possibly induces an increase in H_2_O_2_ level and that PAs pretreatment could provide protection to plants by detoxification of H_2_O_2_ damage.

While it is clear that oxidative stress was the primary source of damage in excess Cu, lignin biosynthesis was noticeably reduced in the presence of excess Cu in this study. Lignin is found in plant secondary cell walls and the biosynthesis and polymerization of lignin require H_2_O_2_, its synthesis is often induced in the presence of pathogens and excess heavy metals [63]. Conversely, lignin was much reduced but was restored by the addition of PAs in our study. The inhibition of lignin biosynthesis here may be due to a number of reasons, primarily cell death by autophagy resulting from the excess Cu levels in this study, as has been reported previously for excess heavy metals, then respiration and photosynthetic disruption to produce energy in the form of ATP and the precursors for lignin biosynthesis, as lignin is the second most abundant biopolymer, and oxidative damage to both protein and lipids would result in the inability to transport the precursors to the cell surface. The addition of a reductant, either AsA or PAs, resulted in partial restoration of many parameters by relieving this oxidative stress and the restoration of lignin biosynthesis. In fact, this appeared to be an overall trend in the treatment with Cu alone, because gene ontology analysis showed that a variety of up-regulated DEGs were enriched in extracellular activities. Moreover, many of these up-regulated genes were greatly reduced by pretreatment with PAs, to the extent that the alfalfa could germinate and survive. Thus, pretreating seeds with reductants or compounds may be an effective strategy to prevent the massive oxidative damage and ensure the establishment of plants in Cu-rich soils that may be toxic to their development. Once established, the plant can carry out a variety of processes, involving lignin biosynthesis to tolerate high levels of Cu.

Besides Cu, a number of other heavy metals can also accumulate in the soil including aluminum, Cd, and zinc. Additionally, a pathogen attached to young seedlings can greatly inhibit the establishment. A common response to all these root stresses appears to be involved in the secondary wall and lignin synthesis combined with increased activity of anti-oxidant stress systems [63]. Thus, Cd tolerance in alfalfa reveals the importance of glutathione-based anti-oxidant systems [64]. Overexpression of glutaredoxin in Arabidopsis reduced the accumulation of heavy metals and increased the level of antioxidant molecules and various physiological parameters [65]. In our study here, the induction of AsA biosynthesis was a notable response to excess Cu, and the tolerance could be enhanced by the addition of PAs. Thus, a combination of coating the seeds with strong anti-oxidants such as PAs prior to sowing and selection of anti-oxidant optimized germplasm will be helpful in improving tolerance to excess Cu and other oxidative stress. Although our assays indicated that exogenous addition of PAs could improve alfalfa tolerance to excess Cu, in accordance with previous reports [31], we suspected that the content of endogenous PAs on the seed coat may also affect the germination of alfalfa. More assays need to be carried out in the future to support our speculations, such as selecting different cultivars of alfalfa and analyze the relationship of PAs content in seed coat and the data of germination and other phenotypes in the excess Cu stress.

AsA biosynthesis occurs in the mitochondria. In this study, the addition of AsA and the induction of the last step of AsA biosynthesis were found to be key aspects. Mitochondria, particularly mitochondrial citrate synthase, aggregation of mitochondria, and mitochondrial programmed cell death are linked to metal toxicity [66–68]. Overexpression of the mitochondrial alternative oxidase alleviates mitochondrial-induced cell death due to aluminum toxicity [69]. Given the central role of mitochondria in REDOX biology, programmed cell death, and stress signalling, optimizing mitochondrial biogenesis and activity would be another strategy to improve the tolerance of plants to heavy metal.

## Conclusion

Excess Cu results in strong oxidative stress that cannot be overcome by plant defense alone. The addition of PAs partially but not totally enables basic plant defense including lignin biosynthesis and anti-oxidant systems, to help plants to overcome this stress, and allow plant germination and seedling growth. Multiple strategies, chemical and genetic, can be deployed to overcome heavy metal tolerance.

## Supporting information

S1 Fig PAs preatment improved seedling growth after exposure to excess Cu treatment. (TIF)

S2 Fig PAs and AsA protect alfalfa seedlings from Cu toxicity by eliminating H_2_O_2_ produced due to excess. (TIF)

S3 Fig Enrichment analysis of biological process, molecular function and KEGG pathway of DEGs under Cu stress and PAs pretreatment. (TIF)

S4 Fig Relative transcript expression levels of antioxidant enzymes in alfalfa cultivars WL656 and SH. (TIF)

S5 Fig Relative transcript expression levels of orthologous genes associated with cell wall in alfalfa cultivars WL656 and SH. (TIF)

S6 Fig Multiple amino acid sequence alignment of four laccase isoforms in *Medicago truncatula* and Arabidopsis. (TIF)

S1 Table Primers used in this study. (TIF)

S2 Table All differentially expressed genes. (XLSX)

S3 Table Differentially expressed genes of cell wall related. (XLSX)

S4 Table Differentially expressed genes of transcription factors. (XLSX)

## References

[1] Andrés-Colás N, Perea-García A, Puig S, Pe Arrubia L. Deregulated copper transport affects Arabidopsis development especially in the absence of environmental cycles. Plant Physiology. 2010; 153: 170–184. doi: 10.1104/pp.110.153676 20335405 PMC2862424

[2] Yruela I. Copper in plants: Acquisition, transport and interactions. Functional Plant Biology. 2009; 36: 409–430. doi: 10.1071/FP08288 32688656

[3] Yamasaki H, Hayashi M, Fukazawa M, Kobayashi Y, Shikanai T. SQUAMOSA promoter binding Protein-Like7 is a central regulator for copper homeostasis in arabidopsis. The Plant Cell. 2009; 21: 347–361. doi: 10.1105/tpc.108.060137 19122104 PMC2648088

[4] Wilson MT. Cytochrome c oxidase: A short review. Inorganica Chimica Acta. 1983; 79.

[5] Mayer AM, Staples RC. Laccase: New functions for an old enzyme. Phytochemistry. 2002; 60: 551–565. doi: 10.1016/s0031-9422(02)00171-1 12126701

[6] Dwivedi UN, Singh P, Pandey VP, Kumar A. Structure–function relationship among bacterial, fungal and plant laccases. Journal of Molecular Catalysis B-enzymatic. 2011; 68(2): 117–128.

[7] Bernal M, Casero D, Singh V, Wilson GT, Grande A, Yang H, et al. Transcriptome sequencing identifies SPL7-regulated copper acquisition genes FRO4/FRO5 and the copper dependence of iron homeostasis in Arabidopsis. The Plant cell. 2012; 24: 738–761. doi: 10.1105/tpc.111.090431 22374396 PMC3315244

[8] Gayomba SR, Jung HI, Yan J, Danku J, Rutzke MA, Bernal M, et al. The CTR/COPT-dependent copper uptake and SPL7-dependent copper deficiency responses are required for basal cadmium tolerance in *A*. *Thaliana*. Metallomics. 2013; 5: 1262–1275. doi: 10.1039/c3mt00111c 23835944

[9] Perea-Garcia A, Andres-Borderia A, Mayo DAS, Sanz A, Davis AM, Davis SJ, et al. Modulation of copper deficiency responses by diurnal and circadian rhythms in *Arabidopsis thaliana*. Journal of Experimental Botany. 2016; 67: 391–403. doi: 10.1093/jxb/erv474 26516126 PMC4682440

[10] Klaumann S, Nickolaus SD, Furst SH, Starck S, Schneider S, Neuhaus HE, et al. The tonoplast copper transporter COPT5 acts as an exporter and is required for interorgan allocation of copper in *Arabidopsis thaliana*. New Phytologist. 2011; 192: 393–404. doi: 10.1111/j.1469-8137.2011.03798.x 21692805

[11] Garcia-Molina A, Andres-Colas N, Perea-Garcia A, Neumann U, Dodani SC, Huijser P, et al. The Arabidopsis COPT6 transport protein functions in copper distribution under copper-deficient conditions. Plant Cell Physiology. 2013; 54: 1378–1390. doi: 10.1093/pcp/pct088 23766354

[12] Fernandes JC, Henriques FS. Biochemical, physiological, and structural effects of excess copper in plants. Botanical Review. 1991; 57: 246–273.

[13] Lara L, Luca S. Copper toxicity in *Prunus cerasifera*: Growth and antioxidant enzymes responses of in vitro grown plants. Plant Science. 2014; 168.

[14] Cota-Ruiz K, Hernandez-Viezcas JA, Varela-Ramirez A, Valdes C, Nunez-Gastelum JA, Martínez-Martínez A, et al. Toxicity of copper hydroxide nanoparticles, bulk copper hydroxide, and ionic copper to alfalfa plants: A spectroscopic and gene expression study. Environmental Pollution. 2018; 243: 703–712. doi: 10.1016/j.envpol.2018.09.028 30228067

[15] Cambrolle J, Garcia JL, Ocete R, Figueroa ME, Cantos M. Growth and photosynthetic responses to copper in wild grapevine. Chemosphere. 2013; 93: 294–301. doi: 10.1016/j.chemosphere.2013.04.080 23746388

[16] Schutzendubel A, Polle A. Plant responses to abiotic stresses: Heavy metal-induced oxidative stress and protection by mycorrhization. Journal of Experimental Botany. 2002; 53: 1351–1365. 11997381

[17] Salt DE, Rauser WE. MgATP-Dependent transport of phytochelatins across the tonoplast of oat roots. Plant Physiology. 1995; 107: 1293–1301. doi: 10.1104/pp.107.4.1293 12228436 PMC157264

[18] Keltjens WG, van Beusichem ML. Phytochelatins as biomarkers for heavy metal stress in maize (*Zea mays* L.) and wheat (*Triticum aestivum* L.): Combined effects of copper and cadmium. Plant and Soil. 1998; 203: 119–126.

[19] Alscher RG, Erturk N, Heath LS. Role of superoxide dismutases (SODs) in controlling oxidative stress in plants. Journal of Experimental Botany. 2002; 53: 1331–1341. 11997379

[20] Mittler R. Oxidative stress, antioxidants and stress tolerance. Trends in Plant Science. 2002; 7: 405–410. doi: 10.1016/s1360-1385(02)02312-9 12234732

[21] Drazkiewicz M, Skorzynska-Polit E, Krupa Z. Copper-induced oxidative stress and antioxidant defence in *Arabidopsis thaliana*. Biometals. 2004; 17: 379–387. doi: 10.1023/b:biom.0000029417.18154.22 15259358

[22] Srivastava S, Mishra S, Tripathi RD, Dwivedi S, Gupta DK. Copper-induced oxidative stress and responses of antioxidants and phytochelatins in *Hydrilla verticillata* (L.f.) Royle. Aquatic. Toxicology. 2006; 80: 405–415. doi: 10.1016/j.aquatox.2006.10.006 17113658

[23] Islek C, Unal BT. Copper toxicity in *capsicum annuum*: superoxide dismutase and catalase activities, phenolic and protein amounts of in-vitro-grown plants. Polish Jouranl of Environmental Studies. 2015; 24(6): 2441–2445.

[24] Liu C, Wang X, Shulaev V, Dixon RA. A role for leucoanthocyanidin reductase in the extension of proanthocyanidins. Nature Plants. 2016; 2: 16182. doi: 10.1038/nplants.2016.182 27869786

[25] Dixon RA, Xie DY, Sharma SB. Proanthocyanidins-a final frontier in flavonoid research? New Phyologist. 2005; 165: 9–28. doi: 10.1111/j.1469-8137.2004.01217.x 15720617

[26] He F, Pan QH, Shi Y, Duan CQ. Biosynthesis and genetic regulation of proanthocyanidins in plants. Molecules. 2008; 13: 2674–2703. doi: 10.3390/molecules13102674 18971863 PMC6245171

[27] Santos-Buelga C, Scalbert A. Proanthocyanidins and tannin-like compounds—nature, occurrence, dietary intake and effects on nutrition and health. Journal of the Science of Food and Agriculture. 2000; 80(7): 1094–1117.

[28] Bagchi D, Bagchi M, Stohs S, Ray SD, Sen CK, Preuss HG. Cellular protection with proanthocyanidins derived from grape seeds. Annals of the New York Acadamy of Science. 2002; 957: 260–270. doi: 10.1111/j.1749-6632.2002.tb02922.x 12074978

[29] Goufo P, Trindade H. Rice antioxidants: Phenolic acids, flavonoids, anthocyanins, proanthocyanidins, tocopherols, tocotrienols, γ-oryzanol, and phytic acid. Food Science and Nutrition. 2014; 2. 10.1002/fsn3.86 PMC3959956 24804068

[30] Xu X, Xie H, Wang Y, Wei X. A-type proanthocyanidins from lychee seeds and their antioxidant and antiviral activities. Jouranl of Agricultural and Food Chemistry. 2010; 58. doi: 10.1021/jf1033202 20964424

[31] Jia LG, Sheng ZW, Xu WF, Li YX, Liu YG, Xia YJ, et al. Modulation of anti-oxidation ability by proanthocyanidins during germination of *Arabidopsis thaliana* seeds. Molecular Plant. 2012; 5: 472–481. doi: 10.1093/mp/ssr089 22115918

[32] Debeaujon I, Leon-Kloosterziel KM, Koornneef M. Influence of the testa on seed dormancy, germination, and longevity in Arabidopsis. Plant Physiology. 2000; 122: 403–414. doi: 10.1104/pp.122.2.403 10677433 PMC58877

[33] Xie DY, Sharma SB, Wright E, Wang ZY, Dixon RA. Metabolic engineering of proanthocyanidins through co-expression of anthocyanidin reductase and the PAP1 MYB transcription factor. The Plant Journal. 2006; 45: 895–907. doi: 10.1111/j.1365-313X.2006.02655.x 16507081

[34] Zhu LJ, Deng XG, Zou LJ, Zhang DW, Lin HH. Enhancement of stress tolerance in cucumber seedlings by proanthocyanidins. Biologia Plantarum. 2017; 61: 323–332.

[35] Peralta-Videa JR, de la Rosa G, Gonzalez JH, Gardea-Torresdey JL. Effects of the growth stage on the heavy metal tolerance of alfalfa plants. Advances in Environmental Research. 2004; 8: 679–685.

[36] Zhou ZS, Wang SJ, Yang ZM. Biological detection and analysis of mercury toxicity to alfalfa (*Medicago sativa*) plants. Chemosphere. 2008; 70. doi: 10.1016/j.chemosphere.2007.08.028 17905409

[37] Sobrino-Plata J, Ortega-Villasante C, Flores-Caceres ML, Escobar C, Del CF, Hernández LE. Differential alterations of antioxidant defenses as bioindicators of mercury and cadmium toxicity in alfalfa. Chemosphere. 2009; 77: 946–954. doi: 10.1016/j.chemosphere.2009.08.007 19732935

[38] Wang SH, Zhang H, Zhang Q, Jin GM, Li ZP. Copper-induced oxidative stress and responses of the antioxidant system in roots of *medicago sativa*. Journal of Agronomy and Crop Science. 2011; 197(6): 418–429.

[39] Jambunathan N. Determination and detection of reactive oxygen species (ROS), lipid peroxidation, and electrolyte leakage in plants. Methods in Molecular Biology. 2010; 639: 292–298. doi: 10.1007/978-1-60761-702-0_18 20387054

[40] Trapnell C, Williams BA, Pertea G, Mortazavi A, Kwan G, van Baren MJ et al. Transcript assembly and quantification by RNA-Seq reveals unannotated transcripts and isoform switching during cell differentiation. Nat Biotechnol. 2010; 28(5):511–5. doi: 10.1038/nbt.1621 20436464 PMC3146043

[41] Bindea G, Mlecnik B, Hackl H, Charoentong P, Tosolini M, Kirilovsky A, et al. ClueGO: a Cytoscape plug-in to decipher functionally grouped gene ontology and pathway annotation networks. Bioinformatics. 2009; 25(8):1091–3. doi: 10.1093/bioinformatics/btp101 19237447 PMC2666812

[42] He F, Machemer-Noonan K, Golfier P, Unda F, Dechert J, Zhang W, et al. The in vivo impact of MsLAC1, a Miscanthus laccase isoform, on lignification and lignin composition contrasts with its in vitro substrate preference. BMC plant Biology. 2019; 19: 552. doi: 10.1186/s12870-019-2174-3 31830911 PMC6909574

[43] Chen H, Zeng Y, Yang Y, Huang L, Tang B, Zhang H, et al. Allele-aware chromosome-level genome assembly and efficient transgene-free genome editing for the autotetraploid cultivated alfalfa. Nature Communications. 2020; 11, 2494. doi: 10.1038/s41467-020-16338-x 32427850 PMC7237683

[44] Shen C, Du H, Chen Z, Lu H, Zhu F, Chen H, et al. The Chromosome-Level genome sequence of the autotetraploid alfalfa and resequencing of core germplasms provide genomic resources for alfalfa research. Molecular Plant. 2000; 13: 1250–1261. 10.1016/j.molp.2020.07.003 32673760

[45] Liu H, Zhang B, Wu T, Ding Y, Ding X, Chu Z. Copper ion elicits defense response in *Arabidopsis thaliana* by activating salicylate- and Ethylene-Dependent signaling pathways. Molecular Plant. 2015; 8(5):1550–3. doi: 10.1016/j.molp.2015.07.008 26225489

[46] Berni R, Luyckx M, Xu X, Legay S, Sergeant K, Hausman JF, et al. Reactive oxygen species and heavy metal stress in plants: impact on the cell wall and secondary metabolism. Environmental and Experimental Botany. 2019; 161: 98–106.

[47] Linster CL, Gomez TA, Christensen KC, Adler LN, Young BD, Brenner C, et al. Arabidopsis VTC2 encodes a GDP-L-galactose phosphorylase, the last unknown enzyme in the Smirnoff-Wheeler pathway to ascorbic acid in plants. Journal of Biological Chemistry. 2007; 282. doi: 10.1074/jbc.M702094200 17462988 PMC2556065

[48] Linster CL, Adler LN, Webb K, Christensen KC, Brenner C, Clarke S. A second GDP-L-galactose phosphorylase in arabidopsis en route to vitamin C. Covalent intermediate and substrate requirements for the conserved reaction. Journal of Biological Chemistry. 2008; 283. doi: 10.1074/jbc.M802594200 18463094 PMC2441562

[49] Glass M, Barkwill S, Unda F, Mansfield SD. Endo-beta-1,4-glucanases impact plant cell wall development by influencing cellulose crystallization. Journal of Integrative Plant Biology. 2013; 57: 396–410. 10.1111/jipb.12353 25756224

[50] Minic Z, Jouanin L. Plant glycoside hydrolases involved in cell wall polysaccharide degradation. Plant Physiology and Biochemistry. 2006; 44: 435–449. doi: 10.1016/j.plaphy.2006.08.001 17023165

[51] Zhang W, Cai C, Staiger CJ. Myosins XI are involved in exocytosis of cellulose synthase complexes. Plant Physiology. 2019; 179: 1537–1555. doi: 10.1104/pp.19.00018 30705068 PMC6446754

[52] Arteca RN, Arteca JM. Heavy-metal-induced ethylene production in *Arabidopsis thaliana*. Journal of Plant Physiology. 2007; 164: 1480–1488. doi: 10.1016/j.jplph.2006.09.006 17215058

[53] Lequeux H, Hermans C, Lutts S, Verbruggen N. Response to copper excess in *Arabidopsis thaliana*: Impact on the root system architecture, hormone distribution, lignin accumulation and mineral profile. Plant Physiology and Biochemistry. 2010; 48: 673–682. doi: 10.1016/j.plaphy.2010.05.005 20542443

[54] Alvarez JP, Furumizu C, Efroni I, Eshed Y, Bowman JL. Active suppression of a leaf meristem orchestrates determinate leaf growth. Elife. 2016; 5. doi: 10.7554/eLife.15023 27710768 PMC5096885

[55] Liu CJ. Deciphering the enigma of lignification: Precursor transport, oxidation, and the topochemistry of lignin assembly. Molecular Plant. 2012; 5: 304–317. doi: 10.1093/mp/ssr121 22307199

[56] Zhao Q, Nakashima J, Chen F, Yin Y, Fu C, Yun J, et al. Laccase is necessary and nonredundant with peroxidase for lignin polymerization during vascular development in Arabidopsis. Plant Cell. 2013; 25: 3976–3987. doi: 10.1105/tpc.113.117770 24143805 PMC3877815

[57] Huang H, Ullah F, Zhou D, Yi M, Zhao Y. Mechanisms of ROS regulation of plant development and stress responses. Frontiers in Plant Science. 2019; 10. doi: 10.3389/fpls.2019.00800 31293607 PMC6603150

[58] Ron M. ROS are good. Trends in Plant Science. 2017; 22. doi: 10.1016/j.tplants.2016.08.002 27666517

[59] Mostofa MG, Hossain MA, Fujita M, Tran LP. Physiological and biochemical mechanisms associated with trehalose-induced copper-stress tolerance in rice. Scientific Reports. 2015; 5. doi: 10.1038/srep11433 26073760 PMC4650698

[60] Dai H, Xu Y, Zhao L, Shan C. Alleviation of copper toxicity on chloroplast antioxidant capacity and photosystem II photochemistry of wheat by hydrogen sulfide. Brazilian Journal of Botany. 2016; 39: 787–793.

[61] Smirnoff N, Wheeler GL. Ascorbic acid in plants: Biosynthesis and function. Critical Reviews in Biochemistry and Molecular Biology. 2000; 35: 291–314. doi: 10.1080/10409230008984166 11005203

[62] Broad RC, Bonneau JP, Hellens RP, Johnson A. Manipulation of ascorbate biosynthetic, recycling, and regulatory pathways for improved abiotic stress tolerance in plants. International Journal of Molecular Sciences. 2020; 21. 10.3390/ijms21051790 PMC7084844 32150968

[63] Dixon RA, Barros J. Lignin biosynthesis: Old roads revisited and new roads explored. Open Biology. 2019; 9(12): 190215. doi: 10.1098/rsob.190215 31795915 PMC6936255

[64] Cui W, Yao P, Pan J, Dai C, Cao H, Chen Z, et al. Transcriptome analysis reveals insight into molecular hydrogen-induced cadmium tolerance in alfalfa: The prominent role of sulfur and (homo) glutathione metabolism. BMC plant Biology. 2020; 20: 58. doi: 10.1186/s12870-020-2272-2 32019510 PMC7001311

[65] Kumar A, Dubey AK, Kumar V, Ansari MA, Narayan S, Meenakshi, et al. Over-expression of chickpea glutaredoxin (CaGrx) provides tolerance to heavy metals by reducing metal accumulation and improved physiological and antioxidant defence system. Ecotoxicology and Environmental Safety. 2020; 192: 110252. doi: 10.1016/j.ecoenv.2020.110252 32014725

[66] Anoop VM, Basu U, McCammon MT, McAlister-Henn L, Taylor GJ. Modulation of citrate metabolism alters aluminum tolerance in yeast and transgenic canola overexpressing a mitochondrial citrate synthase. Plant Physiology. 2003; 132: 2205–2217. doi: 10.1104/pp.103.023903 12913175 PMC181304

[67] Bi Y, Chen W, Zhang W, Zhou Q, Yun L, Xing D. Production of reactive oxygen species, impairment of photosynthetic function and dynamic changes in mitochondria are early events in cadmium-induced cell death in *Arabidopsis thaliana*. Biology of the cell. 2009; 101: 629–643. doi: 10.1042/BC20090015 19453296

[68] Li Z, Xing D. Mechanistic study of mitochondria-dependent programmed cell death induced by aluminium phytotoxicity using fluorescence techniques. Journal of Experimental Botany. 2011; 62: 331–343. doi: 10.1093/jxb/erq279 20937730

[69] Liu J, Li Z, Wang Y, Xing D. Overexpression of ALTERNATIVE OXIDASE1a alleviates mitochondria-dependent programmed cell death induced by aluminium phytotoxicity in Arabidopsis. Journal of Experimental Botany. 2014; 65: 4465–4478. doi: 10.1093/jxb/eru222 24863436

