# Supplementary figures and images for: Exogenous proanthocyanidins improve tolerance of Cu-toxicity by amelioration of oxidative damage and re-programming of gene expression in Medicago sativa
Source: PLoS One. 2021 Oct 26;16(10):e0259100. doi: 10.1371/journal.pone.0259100 (PMC8547628; doi:10.1371/journal.pone.0259100)

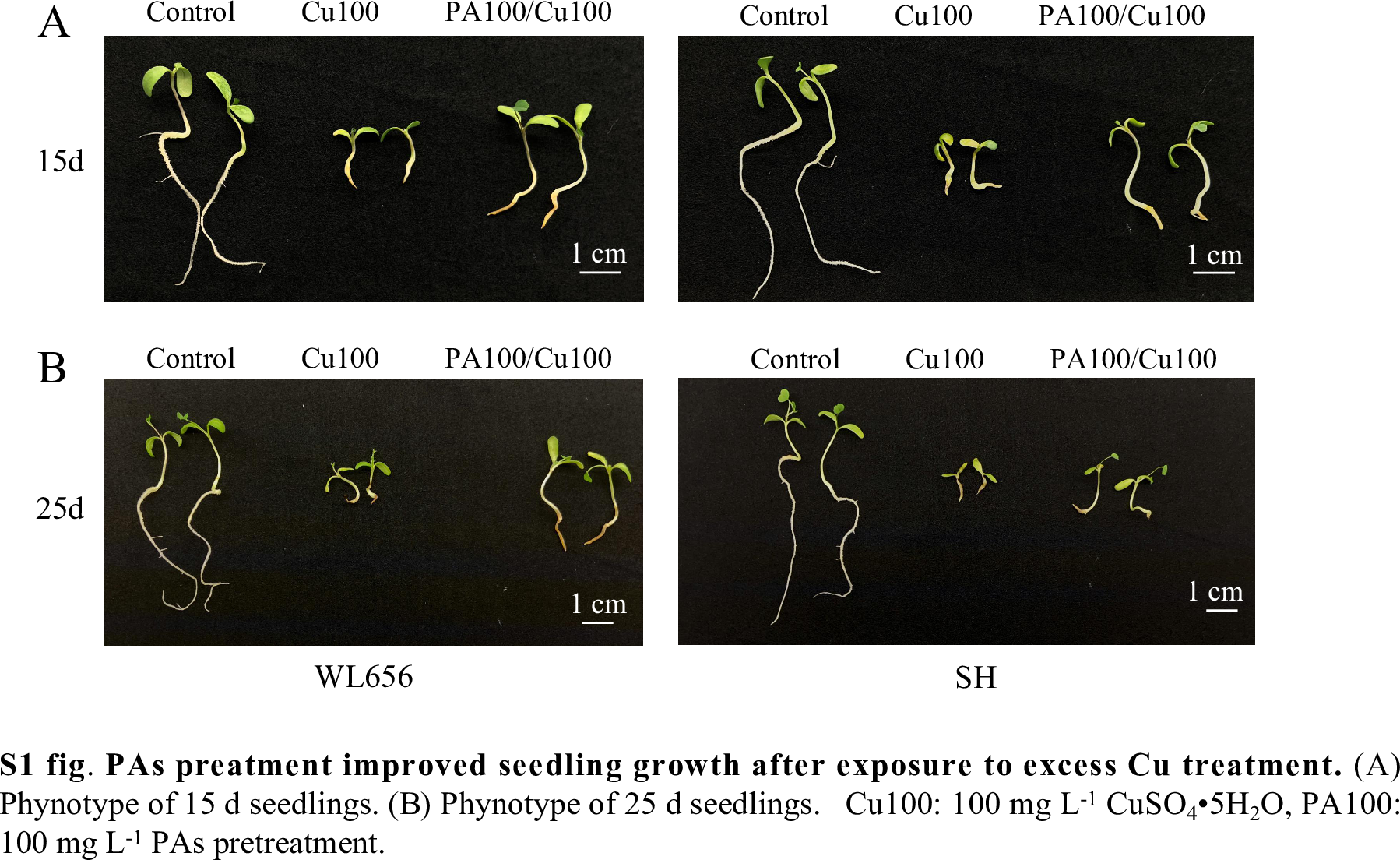

Supplement: S1 Fig — (TIF) [file pone.0259100.s001.tif]

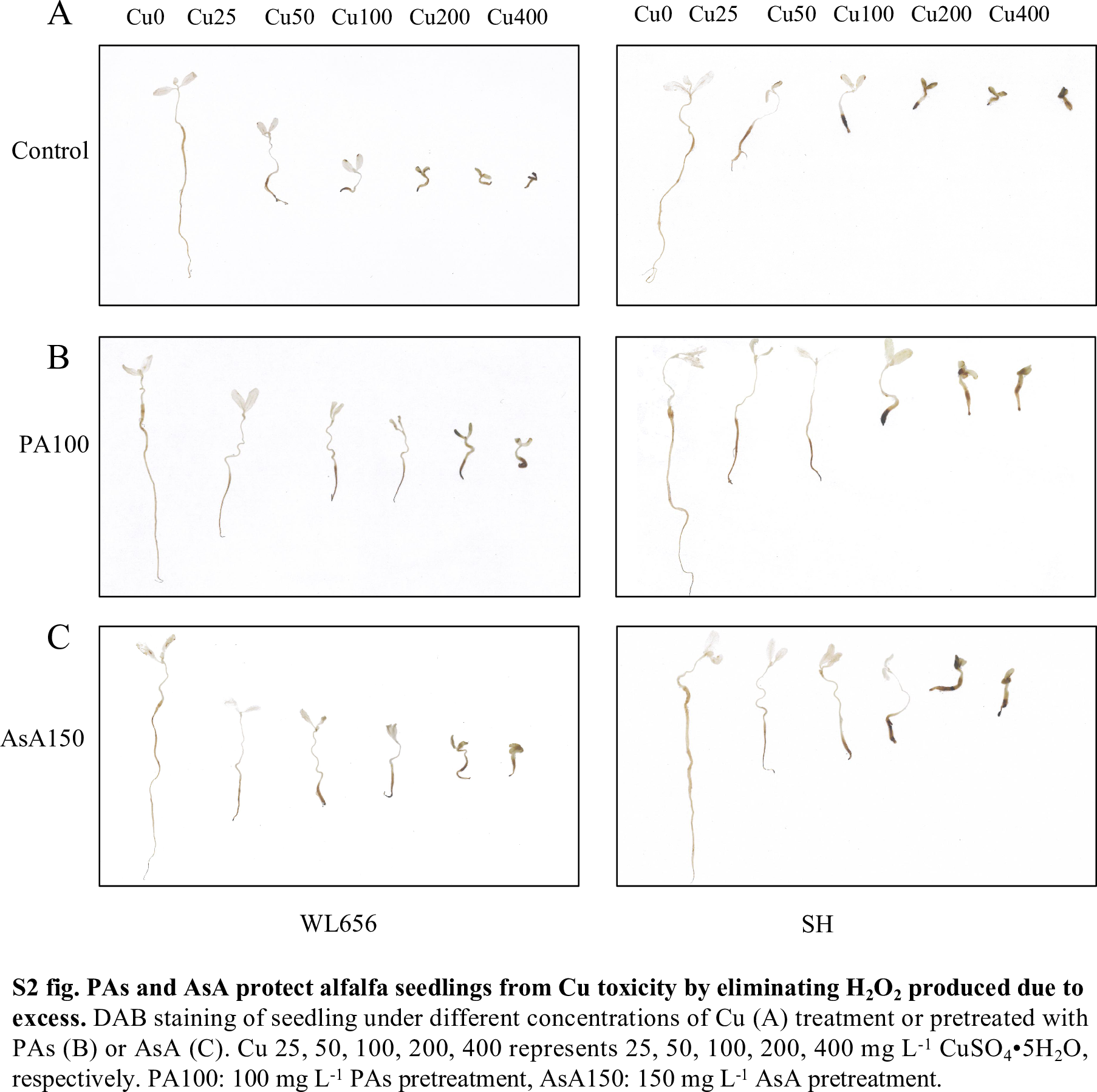

Supplement: S2 Fig — (TIF) [file pone.0259100.s002.tif]

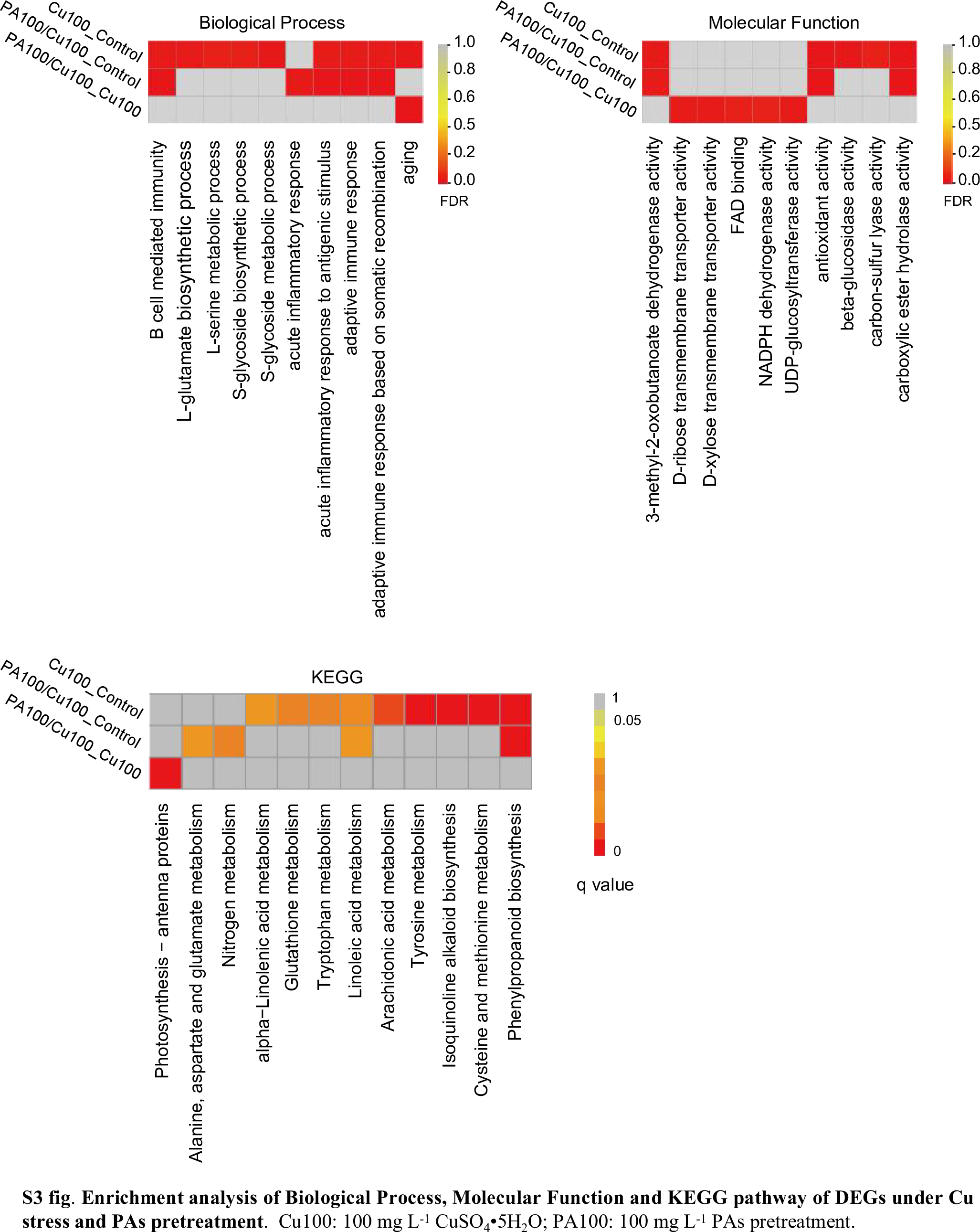

Supplement: S3 Fig — (TIF) [file pone.0259100.s003.tif]

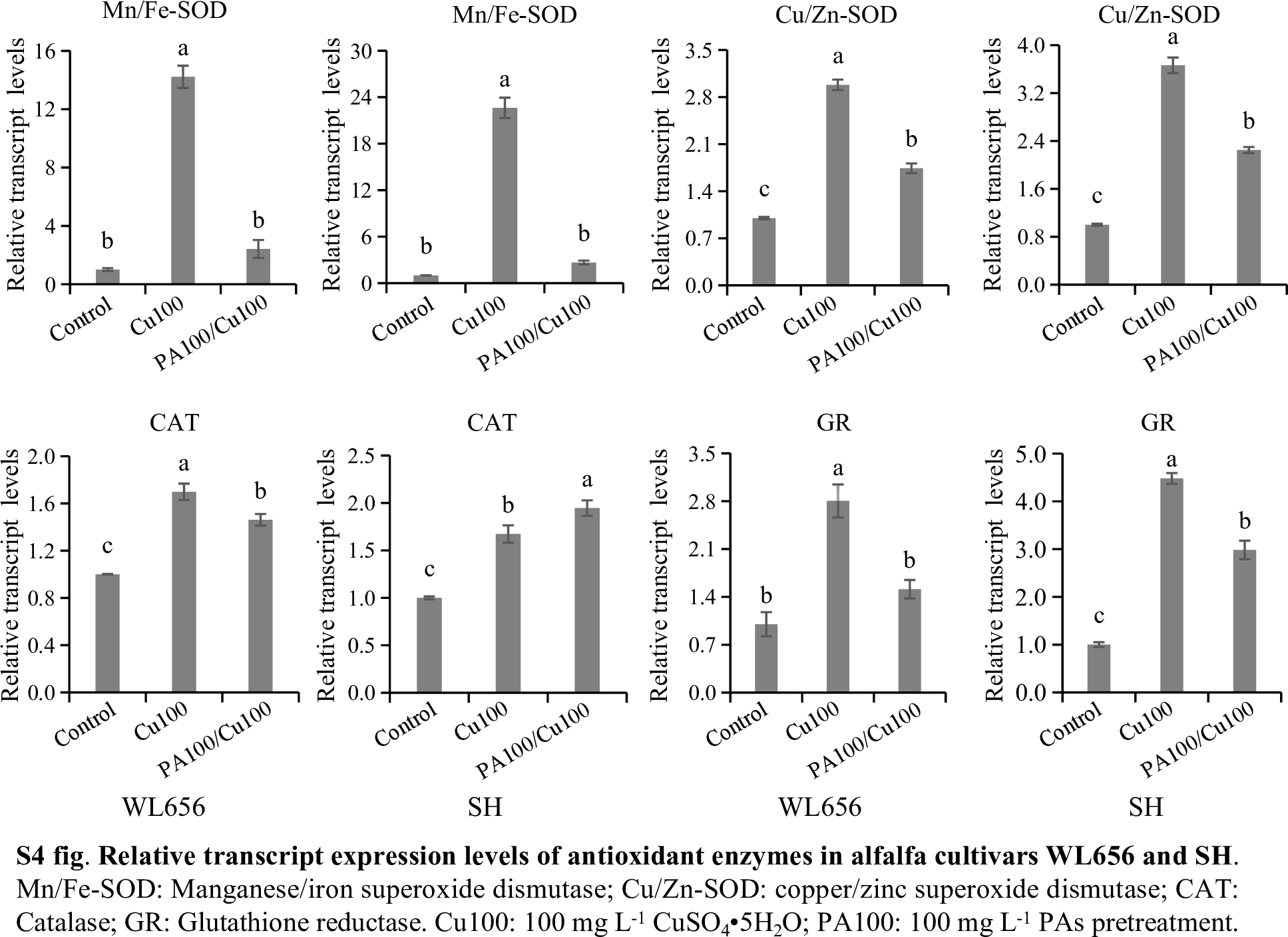

Supplement: S4 Fig — (TIF) [file pone.0259100.s004.tif]

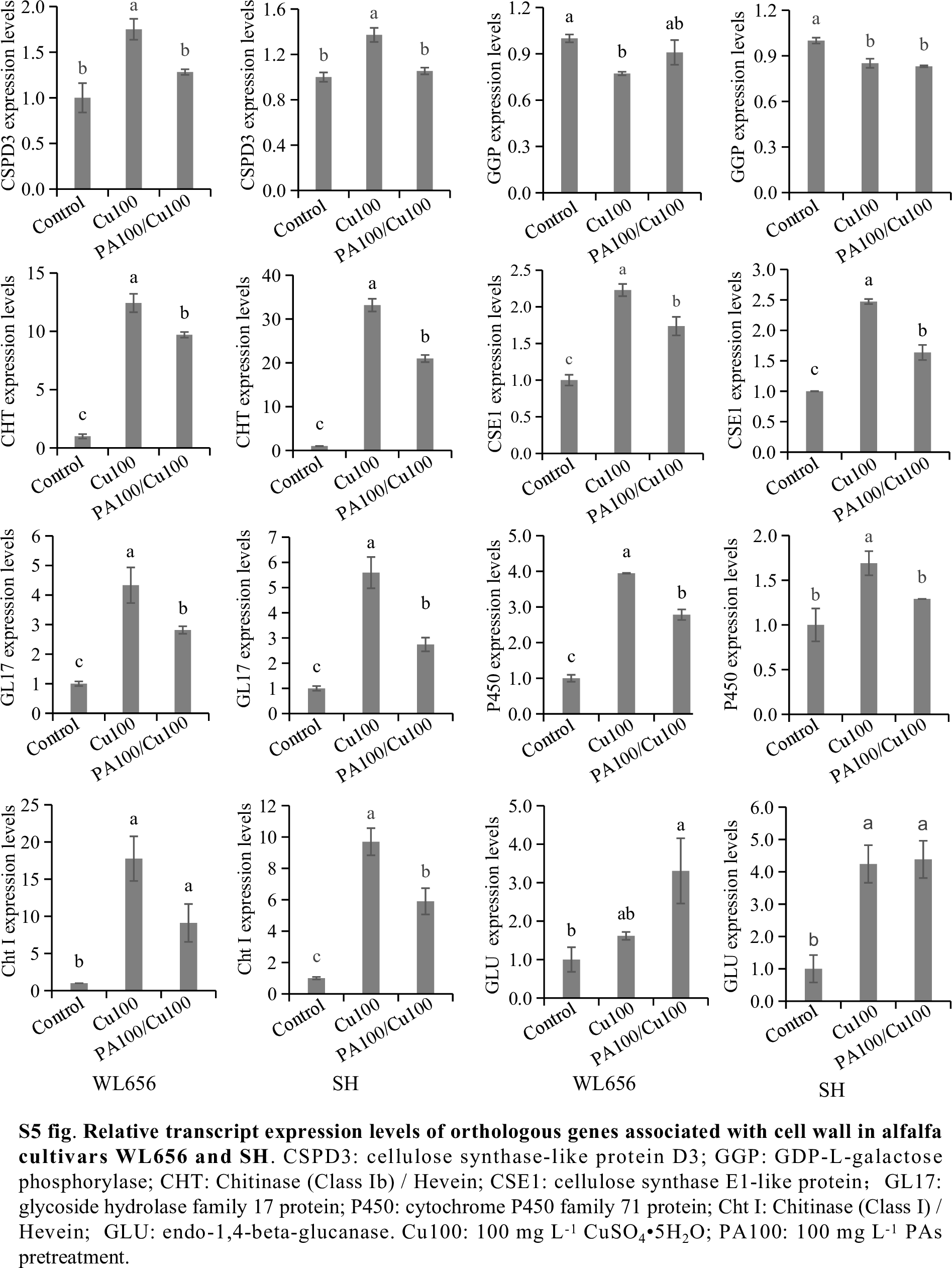

Supplement: S5 Fig — (TIF) [file pone.0259100.s005.tif]

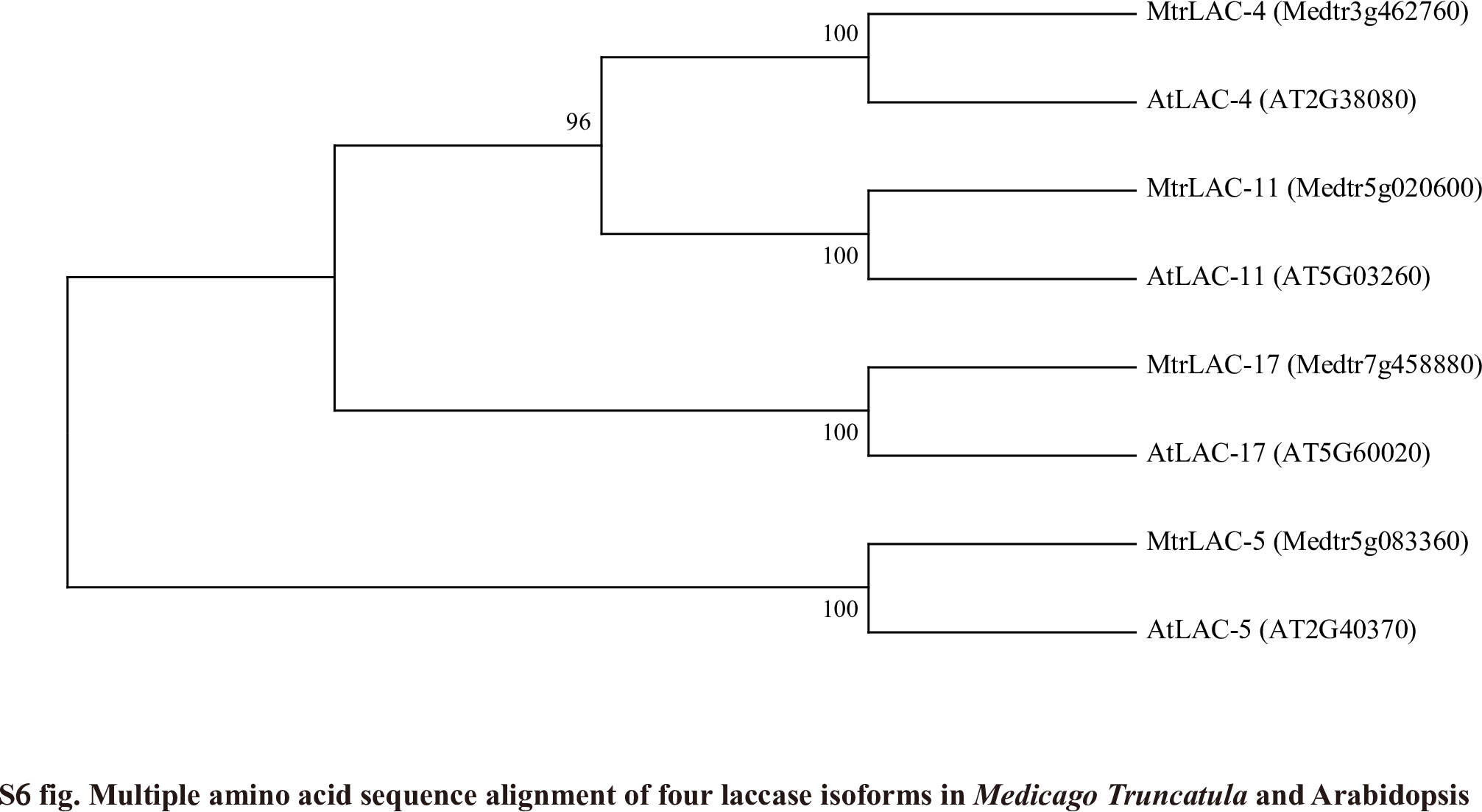

Supplement: S6 Fig — (TIF) [file pone.0259100.s006.tif]

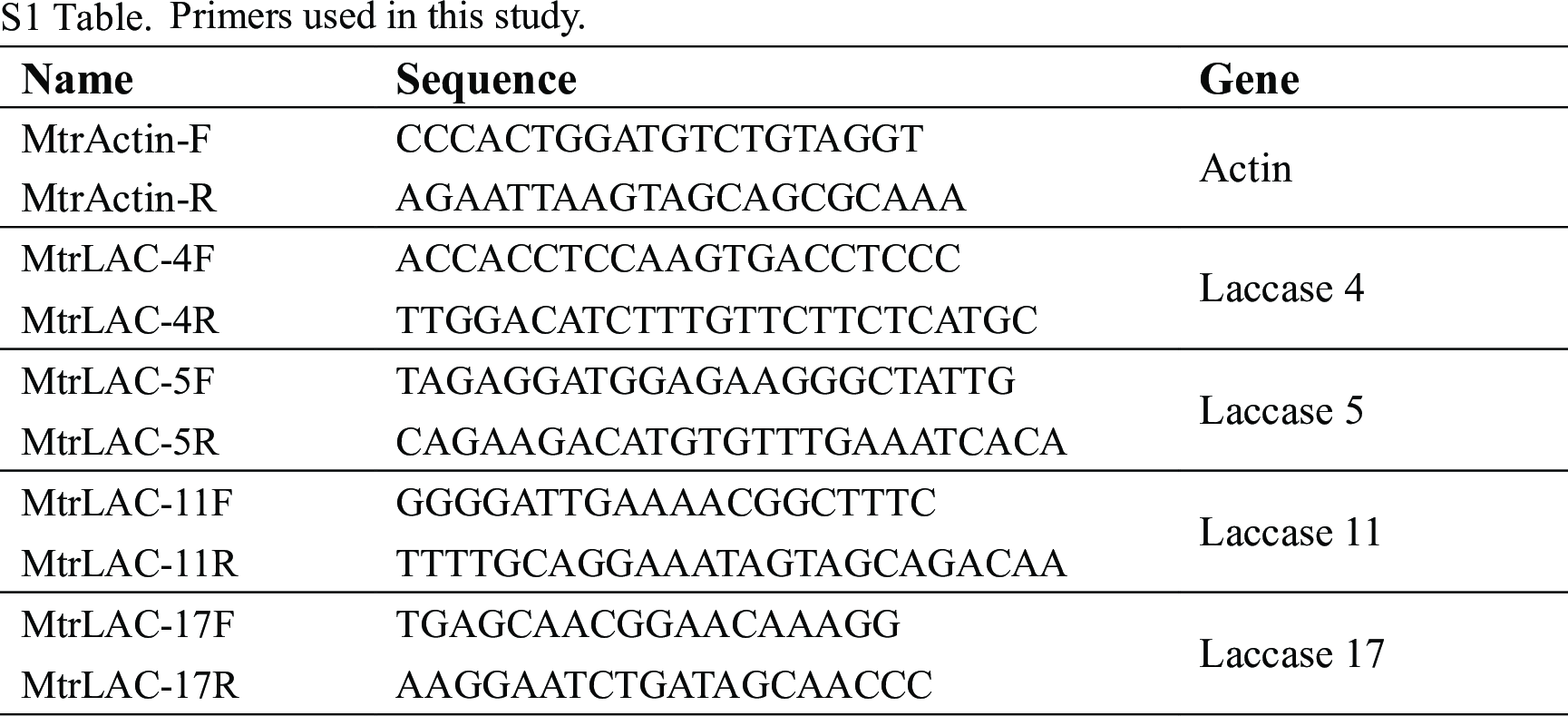

Supplement: S1 Table — (TIF) [file pone.0259100.s007.tif]
